# The mental-attention Tai Chi effect with older adults

**DOI:** 10.1186/s40359-016-0137-0

**Published:** 2016-05-31

**Authors:** Theresa H. M. Kim, Juan Pascual-Leone, Janice Johnson, Hala Tamim

**Affiliations:** School of Kinesiology and Health Science, York University, 4700 Keele Street, Toronto, Ontorio M3J 1P3 Canada; Department of Psychology, York University, Toronto, M3J 1P3 Canada

**Keywords:** Tai Chi, Mental-attention, Working memory, Attentional inhibition, Attentional balance, Field-dependence-independence

## Abstract

**Background:**

Tai Chi practice has some fitness, wellness, and general cognitive effects in older adults. However, benefits of Tai Chi on *specific* mental-attentional executive processes have not been investigated previously. We studied older Canadian adults of Chinese and non-Chinese origin and from low socioeconomic areas.

**Methods:**

Sixty-four adults (51–87 years old) took part in a 16-week Tai Chi program. There were two groups: Chinese-background (*n* = 35) and Non-Chinese-background (*n* = 29). They received four mental-attention executive tasks before and after the 16-week period. These tasks measured visuospatial reasoning, mental-attentional activation (working memory), attentional inhibition, and balance between these attention factors (field-dependence-independence).

**Results:**

Chinese participants showed significant gain on Figural Intersections Task (mental-attentional capacity), Antisaccade (attentional inhibition), and Matrix Reasoning (fluid intelligence measure). Both groups evidenced gain on the Water Level Task (attentional balance).

**Conclusions:**

These gains suggest that Tai Chi can improve mental-attentional vigilance and executive control, when practitioners are sufficiently motivated to pursue this practice, and apply themselves (as our Chinese participants seem to have done). We found that Tai Chi enhanced mental attentional executives in the Chinese sample. The largely negative results with Non-Chinese participants might be explained by less strong motivation and by the relatively short Tai Chi practice period, which contrasts with the prior familiarity with Tai Chi of the Chinese participants.

## Background

Practices that could foster physical and mental fitness in older adults increase in importance as life expectancy advances [1, 2]; not only physical but cognitive functioning may decline with age [3]. Research shows that physical exercise can reduce or reverse such decline in older adults [4, 5], and could lower risk of dementia or delay its onset [6]. Recent meta-analyses of pre-post studies, and randomized controlled trials, find that aerobic exercise increases cognitive functioning (e.g., attentional alertness and working memory) in older adults, without clarifying why [7, 8]. Research has focused largely on aerobic exercise, and there has been less research on meditation-in-movement forms of exercise such as Tai Chi. Tai Chi (also called Tai Chi Chuan or TaiJi) is known for its low-intensity and slow pace, making it most suitable for older persons [9, 10].

Tai Chi is an ancient low-intensity meditative exercise [9], which does not exceed 55 % of maximal oxygen intake [11]. Regularly practiced, it can have potent effects on physical and mental fitness. It is well known that by doing Tai Chi people can achieve calmness – coordinating prescribed movement patterns with mental concentration and slow breathing [12]. Research has shown a positive relationship between Tai Chi practice and executive-driven cognition. Cross-sectional and randomized controlled-trial studies, in Hong Kong and the United States, have found that older people who practice Tai Chi show greater gains in general attention and memory than those practicing Western exercise, such as stretching or dancing [2–4, 13–15].

Why should Tai Chi improve cognitive functioning better than dancing or ordinary gym work? This question is important, because Tai Chi is neither manifestly mental nor does it involve complex cognitive processing – it is a slow motor-sequence ritual. The research literature and a suitable task analysis of mental processes involved in Tai Chi (see our Discussion section) both suggest that its practice induces *mental-attentional vigilance* (mental alertness), and not just ordinary cognitive improvement, in the practitioner – a mental vigilance that may persist for some time between regular Tai Chi sessions. To explain what we mean by mental-attentional vigilance, consider an intriguing repeated finding that researchers have failed to interpret [2, 10, 16]. Taylor-Piliae et al., and others, have found that Backward Digit Span (BDS) but not Forward Digit Span (FDS) shows a gain with Tai Chi relative to a Western exercise practice [2]. These two versions of the digit span task (albeit very similar in content and method) differ in one important respect: in BDS the participants must repeat a sequence of digits in an inverse order. This requires *effortful mental inhibition* of the automatic forward-repeating of the sequence, which is common and more natural in everyday life. Mentally effortful inhibition is a component of *mental/endogenous attention* – the functional-maturational core of working memory [17, 18]. This form of attention is used in working memory and complex executive cognitive processes (fluid-intelligence tasks, etc.) and often is expressed in the brain by activity in the dorsolateral prefrontal lobes [19].

Mobilization of mental attention with effortful inhibition is required when tasks are *misleading* [20–24]. In misleading situations enduring habits (e.g., life-long habits to repeat sequences in a forward manner) or features of the situation can lead participants to error. FDS, in contrast, presents a *facilitating* situation, one that does not present misleading aspects and thus is more easily handled. Tai Chi practice may not have a special beneficial influence in facilitating cognitive tasks. Researchers have found data consistent with this conclusion, but have failed to interpret it appropriately.

Matthews and Williams [25] and others [10] found that Tai Chi practitioners improved, relative to control subjects, in Trail Making Test-B but tended not to differ in performance in Trail Making Test-A. Trails A and B are very similar in content and method. Trails A requires participants to use a pencil to connect, in forward order, a series of numbers (or of letters) randomly spread on a page. Trails B, in contrast, requires to trace forward two sequences in alternation, one of numbers and another of letters (or of yellow numbers versus pink numbers) [15], both mixed and spread randomly over a page. Forward sequences of letters and of numbers are habitual in life activities and are well automatized. This makes Trails A fully facilitating. This habit becomes misleading, however, when alternation is required in Trails B. Thus, *mental-attentional inhibition is necessary to suddenly shift in B from one sequence to the other*. This distinction is recognized in the cognitive-science literature [26], in which B (but not A) is accepted as measure of switching/shifting mental attention and as test of executive functions that activate prefrontal lobes.

Other research shows that for *facilitating* cognitive tasks Tai Chi does not improve performance beyond that of control subjects – although this often is not explicitly recognized [1, 15]. Performance on the Mini Mental State Examination, a basically facilitating cognitive assessment that involves easy information questions etc., often does not improve with Tai Chi practice in ordinary aged people [27, 28].

We call *Tai Chi effect* the apparent aptitude of Tai Chi practice to *improve readiness of mental-attentional vigilance*, that is, the use of mental attention with effortful inhibition, *while at the same time* (as is well recognized but we shall not investigate) *bringing a soothing affective/emotional mood and lowering stress*. The purpose of our study is to investigate with older practitioners whether and why the *Tai Chi effect*, in its specific cognitive improvements, actually happens. To this end we utilize the constructivist-theory procedure called *mental* (or metasubjective) *task analysis* (MTA)*,* a method for theoretical modeling “from within” the subjects’ own processing [22–24, 29–31]. The method helps to infer and understand the mental-attentional processes found in Tai Chi practice. Using this method applied to Tai Chi (see Discussion section) we selected three cognitive tasks, each embodying one of the three key executive activities that our MTA identified in Tai Chi practice.

An MTA of Tai Chi is an operative model of the active executive processes mobilized in participants during Tai Chi practice. Such a model of causal-organismic processes in Tai Chi can lead to quasi-experimental Pre-Post predictions, some of which we test in the current study.

We suggest that three *general-purpose mental-resource factors* enable Tai Chi performances: (1) application of high mental-attentional activation and vigilance – indexed in this study by the visuospatial Figural Intersections Task (FIT); (2) attentional interruption (inhibition) of habitual/automatized processes that interfere with the task – indexed in this study by the well-known Antisaccade task; and (3) dynamic balancing of mental activation and mental inhibition, optimizing flow of valid performance – here indexed by Water Level Task (WLT), a quantitative measure inspired by Piaget’s work on representation of horizontality. Our goal was to examine, in a Pre-Post design, whether Tai Chi practice fosters these specific mental-attentional executive factors, leading to improved performance on our three criterion tasks (i.e., FIT, Antisaccade, and WLT). If it does, this would be strong evidence supporting our prediction of specific mental-attentional processes required in Tai Chi practice. Since these tasks are completely different from Tai Chi in content and method, the increment of their Post-scores following Tai Chi practice would support two theoretical inferences: (1) Tai Chi promotes specific use of mental/executive attention (i.e., the key maturational component of working memory), and (2) these mental-attentional resources are content-free and general purpose – as we, and other cognitive psychologists, have maintained [17, 32, 33].

This is a repeated-measures design to investigate the presence of predicted, specific attentional executive processes in Tai Chi by way of showing that these processes are primed and potentiated by Tai Chi practice. Although other researchers have recognized usefulness of Tai Chi practice for improving cognitive vigilance in middle-aged and older adults, they have offered no explanation for it; and researchers have wondered how this Tai Chi effect occurs [1, 2, 15, 16].

We used two distinct samples, a main one of Chinese participants and another of Non-Chinese participants. We expected that for cultural-motivational reasons, Chinese participants might learn Tai Chi more easily and practice it more than Non-Chinese participants would. Our expectation was that *if the effect exists,* but is weak due to the short Tai Chi practice period, then the Chinese sample should exhibit the effect; but the Non-Chinese sample might not (or might show it more weakly).

Furthermore, *failure* of the Non-Chinese participants *to exhibit an effect* in the Pre-Post testing comparison could serve as evidence contradicting an alternative interpretation to the predicted positive Chinese results: That a significant Pre-Post effect in the Chinese group might reflect learning due to repeated-testing. Any effect simply due to retesting should occur in both groups. Thus negative results with the Non-Chinese sample, should they occur, could be used as a *non-standard control for learning, via repeated testing, in our criterion tasks with the Chinese sample*. Participants in the Non-Chinese sample went through the same 16-weeks of Tai Chi training, as did the Chinese sample, although with a different English-speaking Tai-Chi master. Problems of budgeting prevented us from making the testing period longer and from recruiting separate standard (active or passive) control groups for both Chinese and Non-Chinese samples. These two limitations of the study are important, but cannot affect the predicted interpretation of results if the Chinese sample exhibits the Tai Chi effect and the Non-Chinese fail to exhibit it or do so to a lesser degree. In summary, the study aimed to appraise cognitive effects of Tai Chi practice (the *Tai Chi effect*) with two samples of community-dwelling middle-aged and older participants, from low socioeconomic urban areas.

## Methods

### Participant samples

This study was part of a broader project assessing biological functioning and psychological well-being in older adults; these biological data are not presented here. Participants were community-dwelling adults of at least 50 years of age; they had a low socioeconomic status. We selected a low socioeconomic sample because of the public-health interest of Tai Chi training in this particular age group.

Our Canadians of Chinese origin were previously (culturally) familiar with Tai Chi, but our non-Chinese were less familiar with it. We initially recruited 98 participants for the cognitive testing component of the Tai Chi program. Fifty-seven (58.2 %) were Chinese Canadians and 41 (41.8 %) were non-Chinese (from South America, Caribbean, South Asia, Middle East, Europe, etc.). Of the original samples, 12 (21.1 %) Chinese and 12 (29.3 %) non-Chinese failed to complete the Posttest measures and were lost to follow-up. Elimination of participants who attended fewer than seven Tai Chi sessions reduced the Chinese sample by a further 10 persons. The final sample, thus, was comprised of 35 Chinese (three males; mean age 64.1 years, *SD* = 6.63, range = 52–78), and 29 Non-Chinese participants (seven males; mean age 70.1 years, *SD* = 9.19, range = 52–87). Given the need for commitment to Tai Chi practice and for cognitive testing sessions, the drop-out rate was not unexpected. Both samples came from neighborhoods with low socioeconomic status (SES) in Toronto, Ontario. Testing occurred between February 2011 and April 2012, with participants recruited at community centers, churches, and other public institutions. The neighborhood chosen for the Chinese sample had high Chinese population density and modest SES. The neighborhood of the second sample was ethnically diverse, with a high density of recent immigrants [34]; it was economically similar to the Chinese-sample neighborhood [35]. Participants completed the Physical Activity Readiness Questionnaire [36], a self-screening tool to evaluate their fitness for starting a new exercise program. All participants passed this screening.

### Study design

Classes took place at local Community Centers. Chinese and Non-Chinese samples had their own Tai Chi master speaking their native language (Mandarin and Cantonese versus English, respectively). Participants were taught the *Yang* short-form style of Tai Chi (24 steps). This style incorporates soft, large, slow, open movements. Each class began with 20–30 min of Gi-gong (warm-up to Tai Chi, practicing breath, movement, and motor awareness), followed by Tai Chi (45–60 min). Classes were offered six times per week for 16 consecutive weeks. Participants were asked to attend at least two classes per week, and follow-up calls were made throughout the study to encourage attendance. However, many participants attended fewer than the requested number of classes. Data from those attending fewer than seven Tai Chi sessions were excluded from analysis. Participants completed cognitive tasks before (Pre) and after (Post) the 16-week Tai Chi program. Pre-testing was completed within the week before the start of the Tai Chi program and Post-testing was done within the week after ending the Tai Chi program.

### Cognitive measures

Our three preselected cognitive tasks (i.e., FIT, Antisaccade, and WLT) index, respectively, mental attentional activation, attentional inhibition, and the balance of these resource-factors that are necessary for Tai Chi practice. We also administered a standardized visuospatial task of general fluid intelligence that uses all three functions in coordination, the Matrix Reasoning Task (MR) [37]. It is important to stress that these measures are very well investigated in the cognitive literature. Performance in them is usually little affected by affective factors such as greater or lesser calmness –the calming mood that Tai Chi practice is known to promote.

**Fig. 1:**
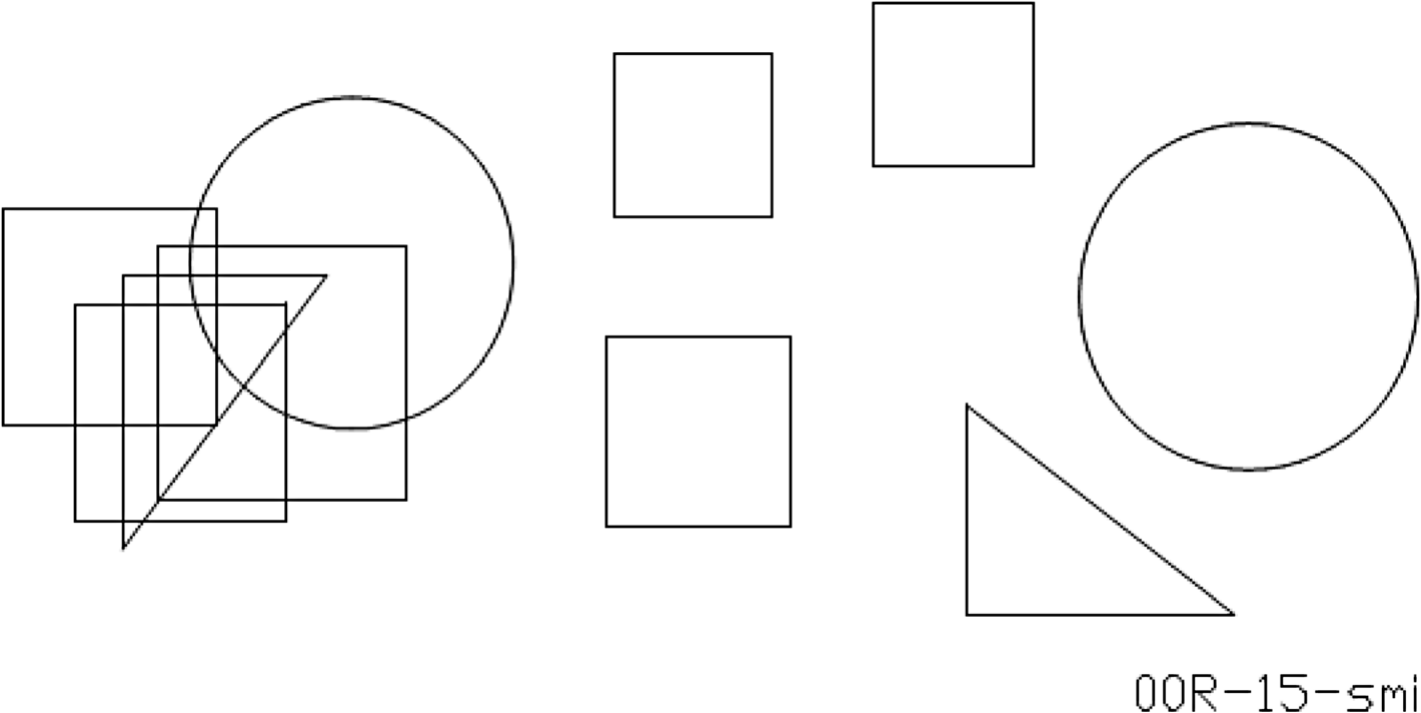
Sample class 5 item from figural intersections task (FIT)

The Figural Intersections Task (FIT) appraises the power of mental activation (i.e., *M-*capacity) of a participant’s mental/endogenous attention [22, 24]. FIT is a paper-and-pen test, with classes of items of graded complexity (2 to 8 shapes presented together). For each item, relevant shapes are presented discretely on the right-hand side of the page, and *their number indexes task difficulty* of the item class in question. The same shapes appear overlapping on the left-hand side (see Fig. 1). Participants must place a mark inside every shape on the right side. Next, they place *a single mark* that is inside all relevant overlapping shapes on the left side (their common intersection). Right and left side shapes may differ in size or orientation, with shape being preserved. Some items contained an *irrelevant* shape on the left to be ignored – irrelevant because it did not appear on the right side. The task contained 36 randomly-ordered items, five in each of classes 2, 3, and 5 through 8; and six in class 4. The score was the total number of correct items. The Pre and Posttest versions of FIT differed only in that items were rotated 180° in the Posttest, which prevents transfer of learning in repeated testing.

**Fig. 2:**
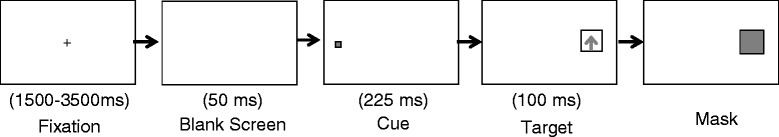
Sample item from antisaccade

The Antisaccade task served to measure attentional inhibition (see Fig. 2). It is used for assessing ability to inhibit the prepotent eye saccade that is prompted by an *orienting reaction* [38–45]. In this computer-based task, a fixation point (+) is presented in the center of the screen for 1500 to 3500 ms (varies randomly), followed by a blank screen for 50 ms. A small solid black square then appears on the left or right side of the screen for 225 ms, immediately followed by a 100 ms target – a stimulus displayed on the opposite side of the screen. This target is replaced by a mask. Target is an arrow pointing up, left, or right, and enclosed in a box-outline. When the black square appears, participants, to succeed, must immediately look to *the side* of the screen *opposite* from the black square, to see the target arrow; they must indicate the arrow’s direction with a keyboard press. Twenty-two practice trials and 90 target trials were administered. Order of stimuli (arrow direction and left vs. right side of screen) was determined randomly for each subject. Score was the proportion of correct target identifications. A split-half reliability coefficient of 0.91 has been obtained for this measure [42]. Antisaccade is a non-verbal task with low memory load [42].

The Water Level Task (WLT) [46, 47] assessed dynamic balance of mental-attention activation and inhibition by appraising performance within the situation. In our paper-and-pen version, items are outlines of rectangular bottles appearing upright, on their side, inverted, or tilted 45°. Each item shows a bottle outline, with instructions to imagine the bottle as capped and half-filled with water. Participants must draw a line marking the top of water and mark an X indicating water location. They completed one practice trial followed by four horizontal/vertical bottles and four (45°) titled bottles, randomly ordered. Each item was scored based on water location (position of ‘X’) and angle of deviation (absolute degrees of deviation of waterline drawn from the horizontal – symbolized on the page by a table line). A high score indicates a more veridical representation, typical of field-independent subjects [48]. Cronbach’s alpha reliability coefficient of 0.86 has been obtained for this task [49], and its validity is documented elsewhere [49–51].

The *Matrix Reasoning* (MR) subtest from *Wechsler Abbreviated Scale of Intelligence* [37, 52] was used to assess non-verbal fluid-intelligence reasoning. MR is a paper-based test in which the majority of items are comprised of a four-cell matrix. Three of the cells contain abstract drawings, and the fourth is empty. The participant must analyze the three drawings to discern a pattern and then select from five choices the appropriate drawing to fill the empty cell. Responses are recorded as correct or incorrect and are converted to an age-adjusted standardized T-score. A higher T-score indicates better psychometric (*g-*factor) intelligence.

Research has shown that performance in FIT (working memory) and Antisaccade (attentional inhibition) decrease with adult aging, as do scores on measures of the general “fluid” intelligence, to which FIT and MR are closely related [23, 24, 53–56].

### Epidemiological variables

Other information collected from participants included number of Tai Chi sessions attended, gender, education, age, annual income, duration of previous Tai Chi, and other physical activity participation.

### Procedure

All cognitive testing was conducted one-on-one, within a 1–2 h period, using Mandarin or Cantonese for the Chinese group, and English for the Non-Chinese group. Order of task administration was: WLT, MR, Antisaccade, and FIT for both Pre and Posttest. In both samples, a few participants completed some but not all measures (often due to fatigue); they were included in analyses of tasks on which they had both Pre and Posttest scores.

### Ethics

The study was approved by the Human Participants Review Sub-Committee, ethics review committee of York University. All participants provided a written consent to participate in the study.

## Results

**Fig. 3:**
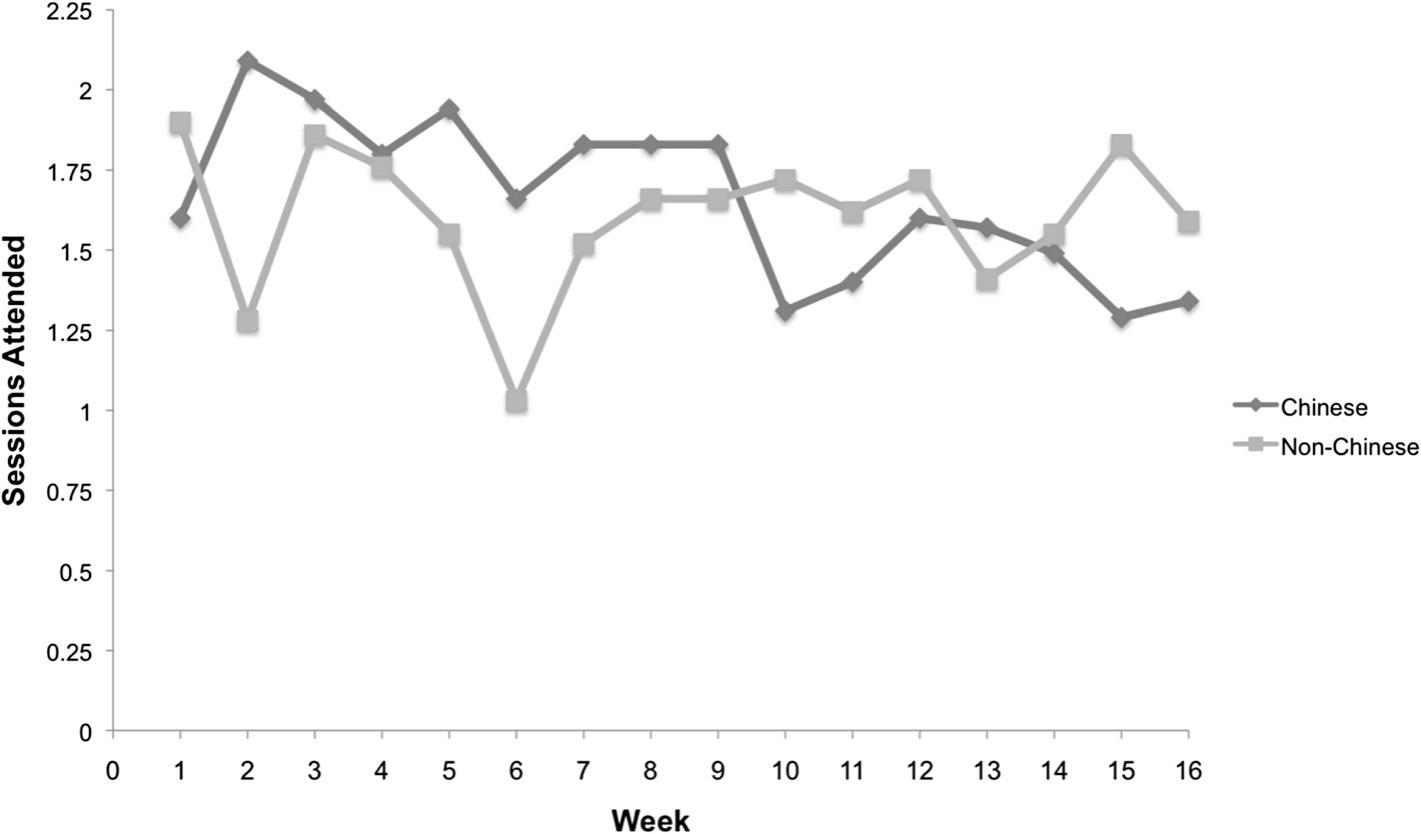
Average weekly Tai Chi attendance for Chinese and Non-Chinese samples

Table 1 summarizes socio-demographic characteristics of participants in the Chinese and Non-Chinese samples. For both groups, the majority were females. The Chinese group was significantly younger than the Non-Chinese (*p* = .004). The Chinese sample was less likely to have at least a high school education and had lower income than the Non-Chinese sample. The majority in both groups reported exercising at least once a week; and when engaged in physical activity, the majority reported making a moderate or intense effort. More Chinese participants had over one year previous experience with Tai Chi. With one extreme outlier removed from each group, the average number of years of previous Tai Chi experience was higher (*p* = .003) for the Chinese sample (*M* = 3.32, *SD* = 4.38) than for the Non-Chinese (*M* = 0.54, *SD* = 1.58). Number of Tai Chi classes attended by Chinese (*M* = 26.54, *SD* = 20.39) and Non-Chinese (*M* = 25.69, *SD* = 12.41) samples was similar (*p* = .844), as was the weekly pattern of attendance (see Fig. 3). Mean Pretest scores on the cognitive measures, for the two samples, appear in Table 2. Despite the noted demographic differences, the two samples did not differ on Pretest scores on any of the cognitive tasks (*p* > .05). Further, participants who were dropped from analyses (due to failure to complete Post-test measures or insufficient number of Tai Chi sessions attended) did not differ significantly from the included participants on Pretest cognitive scores (*p* > .05).

**Table 1:** Demographic characteristics of Chinese and Non-Chinese participants

Demographics	All number (%)	Chinese number (%)	Non-Chinese number (%)	*p*-value
Gender Male Female	10 (15.6) 54 (84.4)	3 (8.6) 32 (91.4)	7 (24.1) 22 (75.9)	.088
Age 50–64 65–74 75+	27 (42.2) 26 (40.6) 11 (17.2)	20 (57.1) 13 (37.1) 2 (5.7)	7 (24.1) 13 (44.8) 9 (31.0)	.006
Education Illiterate Primary Junior/Senior High University	2 (3.3) 21 (34.4) 28 (45.9) 10 (16.4)	2 (5.9) 14 (41.2) 17 (50.0) 1 (2.9)	0 (0) 7 (25.9) 11 (40.7) 9 (33.3)	.010
Income < 14,000 14,000–30,000 > 30,000	34 (59.6) 16 (28.1) 7 (12.3)	25 (75.8) 5 (15.2) 3 (9.1)	9 (37.5) 11 (45.8) 4 (16.7)	.013
Marital Status Unmarried/Widowed/Divorced Married/Living with partner	26 (42.6) 35 (57.4)	11 (33.3) 22 (66.7)	15 (53.5) 13 (46.4)	.111
Previous Tai Chi participation (at least one year) Yes No	22 (34.4) 42 (65.6)	18 (51.4) 17 (48.6)	4 (13.8) 25 (86.2)	.003
Physical activity per week At least once or twice Rarely or never	46 (74.2) 16 (25.8)	23 (67.6) 11 (32.4)	23 (82.1) 5 (17.9)	.088
Intensity of physical activity Intense/Moderate effort Light effort	50 (80.6) 12 (19.4)	26 (76.5) 8 (23.5)	24 (82.7) 4 (14.3)	.357

*p*-value: between-subject *p*-value for Chinese versus Non-Chinese

**Table 2:** Mean scores on cognitive tasks Pre and post Tai Chi training, for Chinese and Non- Chinese samples

	Number	Pre-test		Post-test		Pre vs. Post *p*-value
		Mean	(*SD*)	Mean	(*SD*)	
Chinese sample						
Figural Intersections Task	34	20.06	(8.54)	22.00	(7.96)	.031
Antisaccade	34	.47	(.16)	.58	(.21)	<.001
Water Level Task	35	10.43	(4.34)	11.84	(3.89)	.057
Matrix reasoning	35	42.26	(11.52)	47.17	(11.89)	.002
Non-Chinese sample						
Figural Intersections Task	28	18.86	(6.59)	18.21	(7.48)	.469
Antisaccade	25	55	(.19)	57	(.22)	.628
Water Level Task	28	10.31	(4.96)	12.09	(2.92)	.030
Matrix reasoning	28	44.21	(10.65)	45.04	(11.02)	.525

**Fig. 4:**
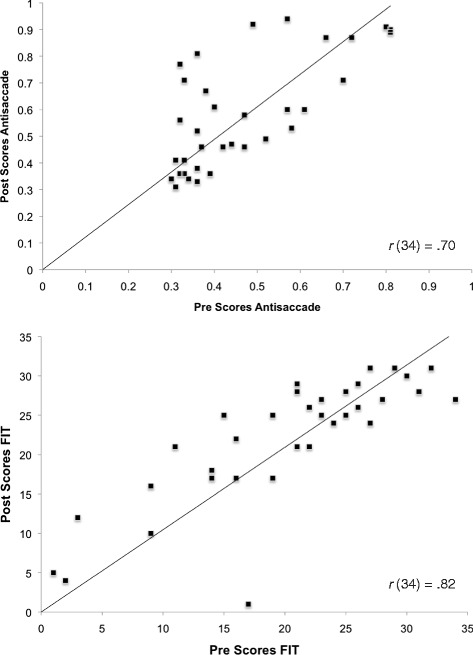
Bivariate plot of pretest vs. Posttest scores on Antisaccade and Figural Intersections Task for Chinese sample

**Fig. 5:**
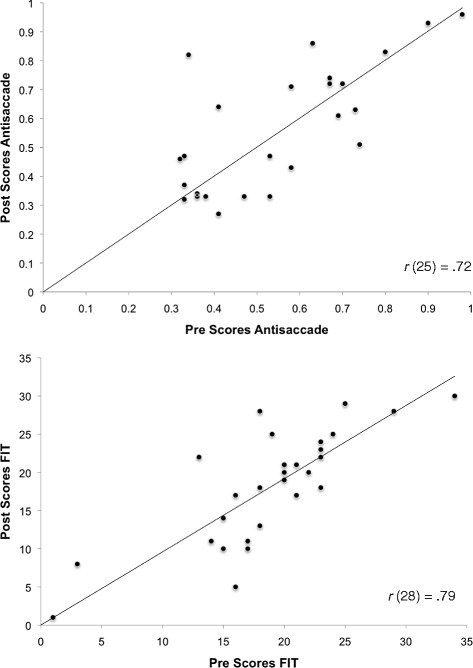
Bivariate plot of pretest vs. Posttest scores on Antisaccade and Figural Intersections Task for Non-Chinese sample

**Fig. 6:**
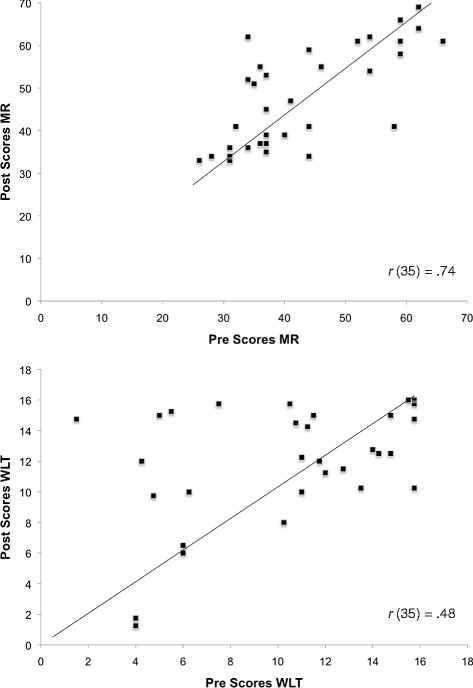
Bivariate plot of pretest vs. Posttest scores on Matrix Reasoning and Water Level Task for Chinese sample

**Fig. 7:**
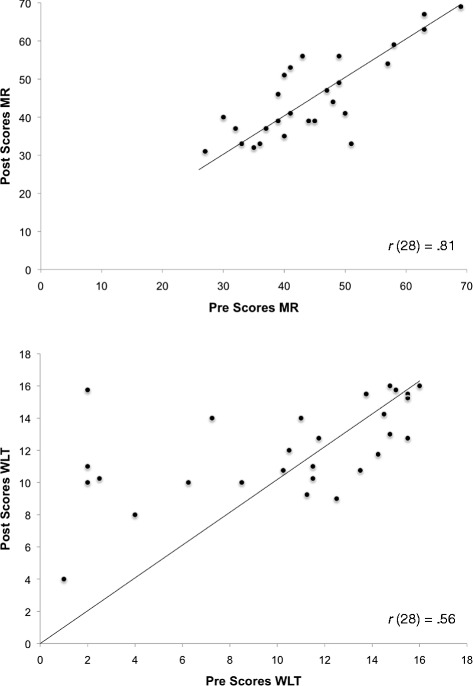
Bivariate plot of Matrix Reasoning and Water Level Task for Non-Chinese sample

Our main interest is in Pre-Post comparison of mean scores on the cognitive measures. To illustrate individual data, we present and emphasize bivariate scatter plots of Pre-versus-Post scores on two key cognitive tasks, Antisaccade and FIT. Figure 4 displays these data for the Chinese sample, and Fig. 5 for the Non-Chinese sample. Figure 4 shows that for Chinese participants, gain in the Post-testing (i.e., points located above the main diagonal) can be found along the whole range of Pre-scores. Some improvement can be noted across the range of scores in the Non-Chinese sample as well (Fig. 5); but here, gain is less consistent. To allow comparison with the other criterion tasks, MR and WLT, their bivariate scatter plots are given in Figs. 6 and 7.

Table 2 shows mean Pre and Posttest scores on the cognitive measures for the two samples. We used a series of Sample x Time mixed analyses of variance to examine possible gain on each cognitive task. Analyses used the Statistical Package for Social Sciences (SPSS, version 23.0). For FIT, there was a Sample x Time interaction, *F*(1, 60) = 4.34, *p* = .042, ***η***_p_^2^ = .07, but no main effect for sample (*p* = .188) or time (*p* = .300). Only the Chinese sample improved from Pre to Posttest, *F*(1, 33) = 5.05, *p* = .031, η_p_^2^ = .13. Antisaccade showed a main effect for time, *F*(1, 57) = 11.37, *p* = .001, η_p_^2^ = .17, and a Sample x Time interaction, *F*(1, 57) = 6.83, *p* = .011, η_p_^2^ = .11. Again, only the Chinese sample improved significantly on the Antisaccade Posttest, *F*(1, 33) = 21.63, *p* < .001, η_p_^2^ = .40. The WLT yielded a main effect for time, *F*(1, 61) = 9.07, *p* = .004, η_p_^2^ = .13, but no interaction, *F*(1, 61) = 0.12, *p* = .728, η_p_^2^ = .002; both samples improved on the Posttest. Finally, Matrix Reasoning showed a main effect for time, *F*(1, 61) = 8.50, *p* = .005, η_p_^2^ = .12, and a Sample x Time interaction, *F*(1, 61) = 4.33, *p* = .042, η_p_^2^ = .07. Again, only the Chinese sample improved following Tai Chi training, *F*(1, 34) = 11.76, *p* = .002, η_p_^2^ = .26. With the exception of the WLT, the Non-Chinese sample did not show gain in any of the cognitive tasks (*p*’s > .40).

Correlations were computed between cognitive-test difference scores (Posttest minus Pretest) and demographic variables that distinguished the two samples (because the *n*s were low, we report correlations with *p* ≤ .10 for the subsamples). Age did not correlate significantly with any difference score in the whole sample or in either of the subsamples. With the two extreme outliers removed, lifetime number of years practicing Tai Chi predicted MR change score in the whole sample, *r*(57) = .41, *p* = .001, and the Chinese sample, *r*( 32) = .42, *p* = .013. Change in MR score also was positively correlated with income category in the Chinese sample, *r*_*s*_ (31) = .48, *p* = .005. Recall that the Non-Chinese sample did not exhibit significant Pre-Post change in MR; however, their MR change score tended toward a negative relationship with income category, *r*_*s*_(21) = −.36, p = .095. WLT difference score correlated positively with income in the full sample, *r*_*s*_ (54) = .31, *p* = .021, as well as the Chinese, *r*_*s*_ (31) = .29, *p* = .102, and Non-Chinese samples, *r*_*s*_ (21) = .36, *p* = .095. Education category was negatively related to Antisaccade difference score in the Non-Chinese sample, *r*_*s*_ (21) = −.44, *p* = .036. Average number of Tai Chi sessions attended per week related only to change in FIT score in the Non-Chinese sample, *r*(26) = .32, *p* = .095. These correlations lower the likelihood that the Tai Chi effect noted in the Chinese sample is due to confounding variables such as repeated-testing with the criterion tasks.

## Discussion

We found that 16 weeks of Tai Chi instruction resulted in improved scores on all cognitive measures for participants in the Chinese sample, but only on the WLT in the Non-Chinese sample. Why should Tai Chi improve cognitive functioning in some people*,* better than dancing or ordinary gym work does? This question is important, because Tai Chi is just a slow motor-sequence ritual. The research literature and a suitable task analysis of the mental processes involved in Tai Chi suggest that its practice induces *mental-attentional vigilance* (mental alertness), not just general cognitive improvement, in the practitioner; and this vigilance may persist for some time between regular Tai Chi sessions.

### A brief mental metasubjective task analysis (MTA) of Tai Chi and other tasks

Tai Chi practice involves precise, slow, *mindful* body movements that maintain dynamic balance. They cause muscle strengthening without muscle growth – particularly in lower limbs and spinal region [9, 12] – and cause a calming of affects with strong reduction of sympathetic nervous system activity [57]. This slow motor action is performed with full mental awareness of the ritualized prescribed sequence. Mental awareness is necessary to avoid errors and omissions. The Tai Chi sequence contains frequent *misleading moments*. Some of its misleadingness is due to similarity among motor moves, which causes errors. Other sources of misleadingness are contradictory tendencies within the task: The need to sustain mental attention (to sort out the exacting movements) *versus* the need to very calmly maintain without distraction the continuous, relaxed, ritualized motor sequence. There also are numerous distractors, including the automatized habit (to be suppressed during Tai Chi) to move in daily life much faster than Tai Chi prescribes, and the tendency to suffer motor or mental tension during learning of this ritual, the tendency to forget the overall sequence when one has to put attention on demanding local steps, etc.

It is now well recognized [20, 23, 24, 58–60] that to cope with misleading/conflict situations people must use executive-driven activation of *mental/endogenous attention* (working memory capacity) and *attentional inhibition*, both of which *decrease* in power with aging [53–56]. Attentional inhibition is a separate resource-factor whose impairment impacts working memory [19, 38, 39, 61]. Practice and familiarity with a misleading situation, using mental-attentional activation and inhibition, might eliminate misleadingness via automatization, turning the task into a *facilitating* one [23, 24, 62]. A *facilitating situation* occurs when no contradictory scheme is elicited – only task-relevant (often automatized) schemes are activated. Although learning is necessary in Tai Chi, automatization is difficult in this practice due to interference among the moves and distractors. For this reason Tai Chi exhibits a *protracted misleadingness*, a certain tendency not to eliminate performance difficulties with practice.

Our mental (metasubjective) task analysis suggests that in practicing Tai Chi one uses the three general-purpose organismic functions of mental attention discussed above: (1) attention-boosting resource called *M-capacity* [20, 23, 24, 32, 62, 63] – a maturational factor in working memory; (2) a resource-factor of *attentional inhibition,* which functions as an interruption operator [23, 38, 39]; and (3) an executive effort in maintaining dynamic *balance* between the *mental activation* of task-relevant schemes and the *attentional inhibition* of unwanted schemes [20, 22, 38]. There are also a few short moments when, with practice, the task has become facilitating, and *automatic/perceptual attention* [63] run by automatized schemes can be used. We now present a brief mental task analysis of the cognitive criterion tasks we selected for this study, to show their process-analytical correspondence with the processes used in Tai Chi practice; which thus can causally explain in part why the Tai Chi practice in a well-motivated Chinese sample led to improvement in Post-test performance on the criterion tasks.

*Figural Intersections Task* (FIT) [22–24] requires participants to first mark all the detached relevant shapes on the right side of the page (see Fig. 1). This subtask is *facilitating* because all schemes elicited in it have perceptual saliency, which helps in the marking of shapes. The main FIT subtask is to find, on the left, the area of common intersection of the now-overlapping shapes. This subtask is misleading, particularly if many relevant shapes overlap, because then the common intersection tends to be confused with the partial intersections of some shapes. To succeed, participants must use mental attention and boost with activation *all* mental schemes expressing relevant shapes. Mental demand of FIT items (i.e., amount of *M-*capacity needed to solve the item) is indexed by the number of relevant shapes to be mentally intersected.

To illustrate further the task’s misleadingness look at Fig. 1. To find the common intersection of overlapping relevant shapes (let their schemes be f1, f2, f3, …f8), active participants generally use an *analytical strategy* (segregation and joint attending to relevant shapes) allocating attention to shapes and matching them from right-side to left-side of the page to identify the common intersection. One shape (e.g., f1) is used as background, and soon gets chunked with the operative/procedural scheme OP in charge of finding common intersection. The item in Fig. 1 thus would require effortfully keeping in mind schemes OP, f2, f3, f4, and f5 [22]. Thus attentional demand of a class of items is given by the number of relevant shapes. The task yields a culture-fair executive attention/executive attention [26, 64].

The resemblance of this task analysis to Tai Chi practice clearly is not in the tasks’ content. Rather it is in the demand for cognitive resources: good Tai Chi practice imposes a number of constraints (corresponding to the shapes f1…f8 of FIT) to be conjointly maintained. The key constraints in Tai Chi are: (1) move with a continuous movement flow; (2) move slowly; (3) anticipate strictly the sequence of moves; (4) after move *i* produce move *i* + 1 of the sequence; (5) be calm and feel deeply the balance in your body movements; (6) keep all body muscles fully relaxed; (7) maintain dynamic body posture – dynamic balance, upright without tension, shoulders always at prescribed distance from ground, etc.; (8) experience the dynamic body feeling, the flow of “body energy” – Chi in Chinese – as it moves inside one’s body in congruence with bodily moves. Applying together during the practice these constraints is analogous in Tai Chi to segregating the shapes in FIT (f1 to f8, in the most difficult FIT items) to find and mark their joint intersection. A major difference, however, is that Tai Chi constraints could be acquired progressively with practice.

Our criterion task for attentional inhibition was the *Antisaccade task,* an established inhibition measure in the cognitive literature [40]. In this task, brief appearance of a black square on one side of the visual field elicits a strong (instinctive) *orienting reaction* (or saccade) that *must be willfully inhibited* to solve the task [41]. This demands the immediate move of gaze to the opposite-side visual field, in order to see and respond to a target stimulus. Again, although this task’s content is very different from Tai Chi, the latter does involve frequent inhibition of misleading aspects, which could foster, as the data suggest, control of inhibition as indexed by Antisaccade.

Piaget’s *Water Level Task* (WLT) serves to appraise *dynamic balan*c*e* between effortful mental-attention used to imagine proper water-line orientation in empty bottles, and the misleading habits, or distractors, which may prevent a realistic *intuitive imagining of water* inside the empty tilted bottles (a common distractor is to imagine the water at the bottle’s bottom, as it is found in most everyday experiences). Because WLT exhibits protracted (i.e., hard to suppress) misleadingness, children and field-dependent adults [48] are propense to draw the water lines more or less parallel to the bottom or walls of rectangular tilted bottles – irrespective of the bottles’ degree of tilt [46, 49, 65].

### Are data consistent with our operative MTA model of Tai Chi practice?

Prior research has shown that Tai Chi practice can improve cognitive performance of older persons, and perhaps slow down their biological aging [66]. The Chinese sample in the current study had significant gains from Pretest to Posttest performance on FIT, Antisaccade, and Matrix Reasoning (a g-factor intelligence measure). Effect sizes were particularly large for Antisaccade and MR. Western exercise and dancing have been shown to be beneficial for cognitive performance [2, 14]; however, these activities *have not* been shown to affect performance in fluid-intelligence reasoning (Raven Matrices) or in a working memory task (e.g., Backwards Digit Span) – two sorts of tasks with executive function demands similar to those of MR, FIT, and Antisaccade.

The cognitive effects we observed could result from the Tai Chi practice – *Tai Chi effect*: a tendency to induce *readiness or priming* of participants’ *mental-attentional vigilance* that might last for several days. This may be the result of *mobilizing attentional resources,* predicted to be content-free and general-purpose – which would explain their transfer from Tai Chi to the very different mental-attentional tasks. Indeed, this gain in vigilance is not explained by learning due to retesting with the cognitive tasks, because the Non-Chinese sample did not show similar gain during follow up. Such retesting interpretation is also unlikely because we reported significant correlations with our epidemiological variables. Nor can it be explained as transfer of learning from Tai Chi to the Post-tasks, because specific executive and action processes in Tai Chi are unrelated to those of the cognitive tasks.

The WLT yielded a main effect for time, with both samples improving in the Posttest. This positive WLT result suggests that the attentional-control balance may improve with Tai Chi training in both our samples, although improvement due to re-testing cannot be discounted here. The Chinese sample demonstrated a clear Tai Chi effect across tasks, after receiving Tai Chi for at most four months – a short period. With a more intensive and longer Tai Chi practice participants in both samples might have exhibited a Tai Chi effect.

**Fig. 8:**
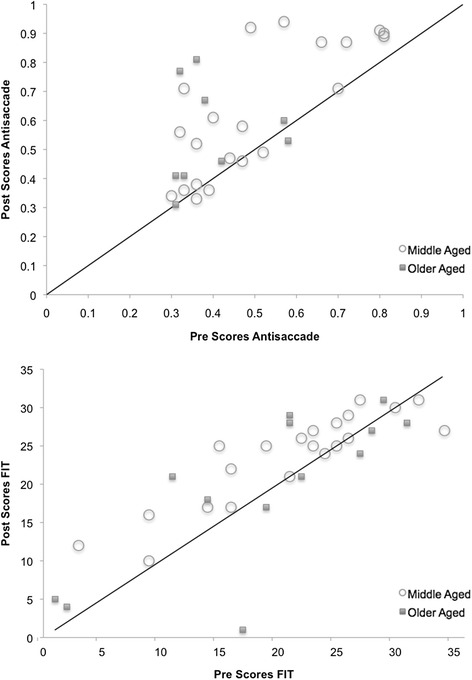
Bivariate plot of pretest vs. Posttest scores on Antisaccade and Figural Intersections Task for Middle-aged vs. Older-aged Chinese participants

The four criterion tasks we used in our study correspond to intelligence processes that in the psychometric literature are called fluid intelligence; and it is very well known that fluid intelligence decreases considerably in old age, in particular after 65 years on the average (e.g., 55, 56). Thus in our attentional-vigilance interpretation of the Chinese results we might expect that the Tai Chi effect would be smaller in old participants than in middle age participants. We have split the Chinese sample into two groups, the middle age participants (between 50 and 65 years of age) and those of old age (more than 65 years). Although our *N* is too small for any certainty, results for Antisaccade and FIT are plotted in Fig. 8. Neither the Antisaccade (*p* = .63) nor the FIT difference score (*p* = .65) differed significantly between the age groups. These data suggest that Tai Chi effect may not significantly change with age, and is still present in old participants.

## Conclusion and limitations of the study

Central attentional inhibition and mental activation – *M-*capacity – are general resources that support people’s capacity for fluid intelligence and working memory, which apply to any content domain. They are used by subjects in developmental and psychometric intelligence, problem solving, social cognition, and emotional intelligence. They are not simple products of learning, but express the brain’s natural endowment in fluid intelligence [17, 19, 23, 24]. Tai Chi practice increases (primes) general availability of these particular mental resources expressed in mental vigilance and readiness for their use. Such is the *Tai Chi effect*. We showed compelling evidence supporting the Tai Chi effect by using four Pre/Post criterion tasks that were carefully selected after *mental task analyses* of both Tai Chi and cognitive tasks. However, Tai Chi practice was only 16 weeks duration, and not all participants attended the recommended two classes per week. Although this short practice did not affect the significant results in Chinese participants, it may be related to largely negative results with the Non-Chinese sample. Replication using longer practice is most advisable.

In our study, due to budgetary limitations and other concerns of the total project (e.g., implementing an exercise program in the community setting), recruitment for proper control groups was not possible. To validate these research results, a replication should be done using random assignment to treatment along with proper control groups, who would receive the Pre-Post tests with some simple interpolated exercise activity (instead of Tai Chi classes). Our samples were comprised mainly of women. Although this does not compromise our predicted finding of a Tai Chi effect, further study with a more gender-balanced sample would also be desirable.

Our findings explain, reinforce, and expand previous results about the possible use of Tai Chi in fostering cognitive readiness. From a Health Science perspective Tai Chi appears as an excellent physical activity for adults (due to the effects on vigilance and executive processing, its effects on affective calmness and fitness, and its very low cost). This Tai Chi practice might be ideal for people with low economic (SES) resources. As Wu Yu-Hsiang, a 19^th^ century Tai Chi Master, said: “your mind should be centered, like the placid cat – peaceful but able to respond instantly [this is vigilance - JPL] to the scurrying mouse” [67].

## Abbreviations

BDS; Backward digit span, FDS; Forward digit span, FIT; Figural Intersections Task, M; mean, M*-*capacity; Mental capacity, MR; Matrix Reasoning, MTA; Mental task analysis , SD; Standard deviation, SES; Socioeconomic status, WLT; Water Level Task.
